# Characterization and transformation of the *CabHLH18* gene from hot pepper to enhance waterlogging tolerance

**DOI:** 10.3389/fpls.2023.1285198

**Published:** 2024-01-12

**Authors:** Huaizhi Tian, Gaoling Fan, Xingwei Xiong, Hui Wang, Suqin Zhang, Guangdong Geng

**Affiliations:** ^1^ College of Agriculture, Guizhou University, Guiyang, Guizhou, China; ^2^ Institute of Pepper, Zunyi Academy of Agricultural Sciences, Zunyi, Guizhou, China; ^3^ Institute of Pepper, Guizhou Academy of Agricultural Sciences, Guiyang, Guizhou, China

**Keywords:** *Capsicum annuum*, CabHLH18, gene cloning, characterization, waterlogging tolerance

## Abstract

Basic helix–loop–helix (bHLH) proteins are important in abiotic stress control. Here, a specific bHLH transcription factor gene, *CabHLH18*, from a strong waterlogging-tolerant pepper cultivar, ‘ZHC2’, was successfully cloned. The *CabHLH18* gene presented a coding sequence length of 1,056 bp, encoding 352 amino acids, and the protein was the closest to *Capsicum annuum* XM016694561.2 protein. The CabHLH18 protein was located in the nucleus. The transformation of the *CabHLH18* overexpression vector into the plumules of hot peppers, ‘DFZJ’ and ‘ZHC1’, exhibited 21.37% and 22.20% efficiency, respectively. The root length, plant height, and fresh weight of the ‘DFZJ’ overexpression lines were greater than those of wild-type (WT) plants under waterlogging conditions. Compared with the WT plants, the overexpression lines generally showed greater contents of water, the amino acid, proline, soluble sugar, root viability, and superoxide dismutase activity, but lower malondialdehyde content under waterlogging conditions. Plant fresh weight, amino acids, proline, and soluble sugar levels of the overexpression lines were 39.17%, 45.03%, 60.67%, and 120.18% greater, respectively, compared with the WT plants at 24 h after waterlogging stress. Therefore, the *CabHLH18* gene could be implicated in conferring waterlogging tolerance in hot peppers and holds promise for enhancing their overall waterlogging tolerance.

## Introduction

*Capsicum annuum* of the Solanaceae family is a vegetable crop of worldwide importance. In 2020, global production was approximately 39.28 million tons (FAO, 2020). Waterlogging is a major abiotic stress that affect plants (Mickelbart et al., 2015). Waterlogging obviously decreases crop production by 32.9% on average (Tian et al., 2021). The annual economic loss is more than billions of dollars (Sauter, 2013; Voesenek and Bailey-Serres, 2015). Waterlogging stress causes physiological and biochemical changes in plants, leading to inhibition of growth and development (Perata and Voesenek, 2007; Bailey-Serres and Voesenek, 2008; Vidoz et al., 2010; Phukan et al., 2016). Under waterlogging stress, the inhibition of plant aerobic respiration limits energy metabolism, thus restraining plant growth and development, including seed germination, vegetative growth, and reproductive growth (Pan et al., 2021). Plants also respond to waterlogging stress by regulating their morphology, energy metabolism, hormone biosynthesis, and signal transduction (Shinozaki and Yamaguchi-Shinozaki, 2007; Hirabayashi et al., 2013; Kuroh et al., 2018). Pepper is a shallow root plant with weak roots and poor waterlogging resistance and can die after a few hours under water, seriously affecting the yield and quality (Molla et al., 2022). Consequently, increasing research to improve the waterlogging resistance of peppers has been conducted. At present, research on waterlogging stress has been focused on wheat, rice, and corn. Most of the studies related to waterlogging stress in peppers have concentrated on morphological observations, physiological examinations, and biochemical analyses, devoting limited attention to waterlogging tolerance mechanisms (Kato et al., 2020; Kaur et al., 2021; Komatsu et al., 2022). Basic helix–loop–helix (bHLH) proteins play important roles in regulating plant resistance to stress, which belong to a superfamily of regulatory proteins present in eukaryotes, having highly conserved bHLH domains (Toledo-Ortiz et al., 2003; Upadhyay et al., 2018). The HLH domain is located at the carboxyl-terminus and consists of two hydrophobic residues in a helical–ring–helical structure, which promotes protein–protein interactions (Murre et al., 1989). bHLH proteins regulate plant growth and development, and biological and abiotic stress responses by suppressing or activating the expression of related downstream genes through transcriptional regulation or nuclear localization. Many bHLH transcription factor genes have been identified from different plants including *Arabidopsis* (Bailey et al., 2003). Zhang et al. (2020) identified 122 members of the bHLH transcription factor family in peppers, among which a few were noted to be involved in responses to cold, heat, drought, and salt stress (Zhang et al., 2020). Low temperature stress can induce significant upregulation of *WbHLH046* gene expression in wheat and improve the expression of the rice *bHLH* gene (*RsICE1*) (Man et al., 2017). *Arabidopsis bHLH122* positively regulates drought tolerance, salt tolerance, and osmotic signaling (Liu et al., 2013), and *CdICE1* of the chrysanthemum bHLH family regulates tolerance to low temperature, drought, and salt stress (Chen et al., 2012). Pepper bHLH transcription factor CabHLH035 can enhance salt tolerance by regulating ion homeostasis and proline biosynthesis (Zhang et al., 2022), and *OrbHLH18* overexpression in *Arabidopsis* can significantly improve cold resistance (Li et al., 2010). The *CsbHLH18* gene of sweet orange enhances cold tolerance in transgenic tobacco (Geng and Liu, 2018). *MebHLH18* expression can increase peroxidase activity, decrease reactive oxygen species (ROS), and change the abscission rate of cassava leaves at low temperatures (Liao et al., 2023). However, no studies have been reported on the waterlogging tolerance effects of the *bHLH* gene in plants.

*bHLH* genes are involved in regulating plant tolerance to abiotic stresses, such as drought, salinity, and low temperature. However, there are no reports on the *bHLH* gene function in hot pepper under waterlogging stress. In the present study, an important bHLH transcription factor gene, *CabHLH18*, in hot pepper was successfully cloned. Next, the sequence characteristics, evolutionary relationship, expression pattern, and subcellular localization of the *CabHLH18* gene were examined. Subsequently, the growth and physiological response of *CabHLH18* overexpression lines under waterlogging stress were analyzed, and the function of the *CabHLH18* gene was preliminarily explored. The results provide a reference for generating waterlogging-tolerant peppers.

## Materials and methods

### Plant materials and treatment

Three hot pepper cultivars, ‘ZHC2’ (waterlogging-tolerant), ‘ZHC1’, and ‘DFZJ’ (waterlogging-sensitive) were used in this study. ‘ZHC2’ and ‘ZHC1’ are inbred lines, which were donated by the Zunyi Academy of Agricultural Sciences (Zunyi, China), and ‘DFZJ’ is a local inbred line from Guizhou province. Pepper seedlings were planted in plastic pots (length × width × depth: 32 × 24 × 13 cm, with one seedling per pot) filled with sand and cultured at 25 ± 2/20 ± 2°C for a 10-h/14-h light/dark photoperiod with an irradiance of 270 μmol m^−2^ s^−1^. Before and after the formation of two leaves, the pepper seedlings were watered with 1/2 Hoagland solution (only macroelements halved, while microelements were not) and further Hoagland solution once a day. After the formation of five leaves, seedlings with uniform growth were transferred to trays for waterlogging treatment and placed into 2-cm-deep water above the sand surface. The seedlings were subjected to three treatments as follows: 6 h (T1) and 24 h (T2) of waterlogging stress and 1 h of recovery (R) after 24 h of waterlogging stress according to the preliminary experiments. Normal culture (no waterlogging stress) conditions served as the control (CK). At each stage, leaf, stem, and root samples were selected from 10 plants, mixed, immediately frozen in liquid nitrogen, and stored in a −80°C freezer until use for gene cloning. The phenotype and physiology of T_3_ pure transgenic hot pepper ‘DFZJ’ and wild-type (WT) plants were determined and cultured as mentioned earlier. Three biological replicates with 10 plants per replicate were established for the experiments.

### RNA reverse transcription, *CabHLH18* amplification, and construction of overexpression vector

RNA was reverse-transcribed into cDNA using a PrimeScript RT kit (Takara, Dalian, China). The full-length coding sequence of the *CabHLH18* gene was amplified from the hot pepper ‘ZHC2’ (under 24 h of waterlogging stress) cDNA, using primers with *Bsa*I restriction sites at the 5′- and 3′-ends. The primers used for amplification are shown in **Table 1**. The amplified fragment was digested with *Sac*I/*Spe*I and *BamH*I/*Kpn*I and inserted into the pEGOEPubi-H vector (modified to contain the green fluorescence protein (*GFP*) gene), using T4-DNA ligase (Takara), according to the manufacturer’s protocol. The inserted sequence was driven by a corn UBI promoter.

**Table 1:** Primers used in this study.

Primer role	Primer sequence (5′–3′)
*CabHLH18* amplification	ActagggtctcGcaccATGGAATATTATGGCTTTAATCAACAATGG ActagggtctcTcgccTATAACCATTTTGAGAGCTGTGTGCAA
PCR identification of transformant	TTAGCCCTGCCTTCATACGC GACACGCTGAACTTGTGG
Control *18S*	TCGGGATCGGAGTAATGA TTCGCAGTTGTTCGTCTT

### Bioinformatics analysis of the *CabHLH18* gene

The *CabHLH18* gene sequence was compared using the DNAMAN global alignment method (Tsai et al., 2006). The NCBI Open Reading Frame (ORF) Finder was used to analyze the ORF of the *CabHLH18* gene and predict its amino acid sequence. Expert Protein Analysis System (Expasy) (https://web.expasy.org/protscale/) was used for hydrophobicity prediction, and NetPhos 2.0 (http://www.cbs.dtu.dk/services/NetPhos/) and CPHmodels 3.2 (http://www.cbs.dtu.dk/services/CPHmodels/) were employed for phosphorylation site analyses. The homologous sequences of the CabHLH18 proteins were retrieved by BLAST search in the NCBI database (accession numbers in **Supplementary Table S1**). A phylogenetic tree was constructed using MEGA 7 (Mega Limited, Auckland, New Zealand) with maximum-likelihood method of 1,000 bootstraps (Kumar et al., 2016). MODELLER9.22 (https://salilab.org/modeller/) was adopted for homology modeling of the CabHLH18 protein (Benjamin and Andrej, 2016), using the X-ray crystal structure of a putative bHLH protein, 5gnj.1.A, as the template. The predicted model was analyzed using SAVES (https://servicesn.mbi.ucla.edu/SAVES/). GROMACS software (http://www.gromacs.org/) was employed for calculating root-mean-square deviation and the potential energy value of the model protein (Hess et al., 2008). Ramachandran plots were examined using Rampage server (http://mordred.bioc.cam.ac.ukrapper/rampage.php) (Lovell et al., 2003).

### Subcellular localization of CabHLH18 protein

Subcellular localization of CabHLH18 protein was analyzed after transient expression in *Nicotiana benthamiana* leaf epidermal cells. The plasmid containing the target gene was amplified and spliced to the 1300-GFP vector by seamless cloning methodology. *Agrobacterium* GV3101 containing the 1300-*CabHLH18*-GFP vector plasmid was cultured. The bacterial cells were suspended in 10 mM MgCl_2_ buffer to an optical density of 600 nm (OD_600_) of 1.0. Two microliters of 100 mM 2-morpholino ethane sulfonic acid was added to the bacterial suspension, which was then incubated for more than 3 h. Subsequently, the prepared bacterial suspension was inoculated into the lower epidermal layer of 3–4-week-old *N. benthamiana* leaves and incubated for 72 h for transformation. As the control, leaf transformation of the 1300-GFP vector with no target gene was used. After transformation and 20 min of 1 µM 4′-6-diamino-2-phenylindole (DAPI) staining, the samples were observed using laser confocal fluorescence microscopy (FV1000 Olympus Corp., Tokyo, Japan). The excitation and emission spectra used for DAPI were 405 nm and 455–470 nm, respectively. For GFP analyses, the excitation and emission spectra were 488 nm and 507 nm, respectively. At least three fields of view from three leaves were examined.

### Gene transformation and identification

*Agrobacterium rhizobiae* strain LBA4404 containing the *CabHLH18* gene was inoculated onto Luria broth (LB) solid medium (with 20 mg/L rifampicin and 50 mg/L kanamycin sulfate) and incubated in the dark for 24 h at 28°C. Then, single colonies were selected and inoculated into LB medium for 24 h under constant shaking. Subsequently, 50 μL of the bacterial culture was inoculated into 50 mL of fresh LB medium and incubated in a shaker (180 rpm, 28°C) for 12 h until OD_600_ of 0.5–0.6 was reached.

After germination of the seeds of hot peppers ‘DFZJ’ and ‘ZHC1’ to a radicle length of 1–2 mm, the seed coats were removed to expose their plumules and placed into the suspension of *Agrobacterium* containing the *CabHLH18* gene, with 200 μL/L SILWET1-77 surfactant and 1 mL/L acetoeugenone under 15 kPa pressure for 5 min. Then, the bacterial suspension was removed, and the seedlings were placed in a clean dish, cultured in dark for 3 days at 28°C, and planted in cell trays with peat substrate. Three biological replicates with 100 seeds per replicate were employed for germination and gene transformation. The T_0_ transgenic seedlings containing the *GFP* reporter gene were detected at the cotyledon stage using a hand-held lamp (LUYOR-3415RG, Shanghai, China). The leaves of the three-leaf stage putative transformants were identified by multiple PCR with specific primers for the UBI promoter and *GFP* gene, and primers for the housekeeping gene *18S* were used as an internal control (**Table 1**). The PCR products were separated on 1% (w/v) agarose gels.

### Analysis of phenotypes and physiological indicators

The T_3_ lines were segregating after self-fertilization of T_0_ transgenic plants. The T_3_ pure overexpression lines of hot pepper were screened by PCR and hygromycin tolerance. The T_3_ lines of hot pepper and WT plants were cultured using the abovementioned method. After the growth of five leaves, samples were collected after 0 h (CK), 6 h (T1), and 24 h (T2) of waterlogging stress, and at 1 h (R1) after recovery. The primary root length, seedling height, fresh weight, water content, and root viability (CAS No. G0124F, Geruisi, Suzhou, China); root proline content (CAS No. BC0295, Solarbio, Beijing, China); and amino acid (not including proline and hydroxyproline, CAS No. BC1575, Solarbio), soluble sugar (CAS No. BC0035, Solarbio), and malondialdehyde (MDA; CAS No. BC0025, Solarbio) levels, and superoxide dismutase (SOD; CAS No. BC0175, Solarbio) activity were analyzed according to the instructions provided in the respective kits. Plant water content was calculated as: plant fresh weight − plant dry weight)/plant fresh weight × 100%.

### Statistical analyses

Statistical software (SPSS 20.00, IBM Inc., Armonk, NY, USA) and graphics software (Origin 2017, OriginLab Inc., Northampton, MA, USA) were used for the data analysis and figure construction, respectively. The Duncan’s multiple range test was performed to determine significant differences between means at a significance level of *p* < 0.05, after showing a significant effect using one-way analysis of variance.

## Results

### Cloning and bioinformatics analysis of the *CabHLH18* gene

**Figure 1:**
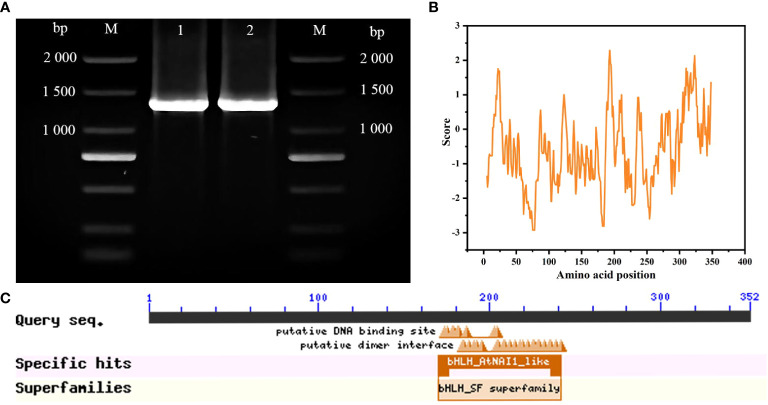
Molecular identification of *CabHLH18* gene in ‘ZHC2’. **(A)** Amplification of bands using ‘ZHC2’ cDNA as template. M: 2,000-bp DNA marker; 1-2: ‘ZHC2’ cDNA. **(B)** Hydrophilic analysis of CabHLH18 protein. **(C)** CabHLH18 protein domains.

**Figure 2:**
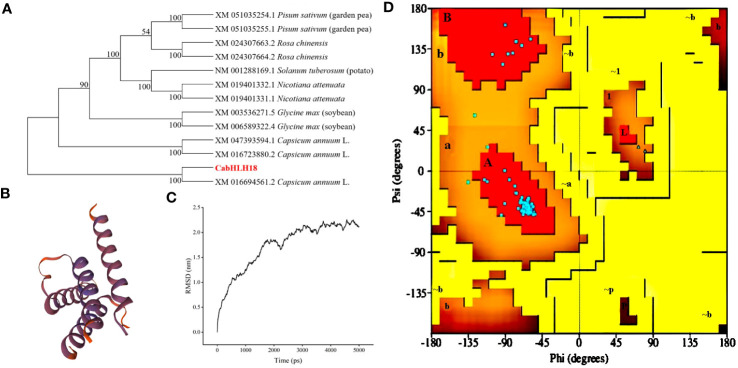
Phylogenetic tree, homology modeling, and molecular simulation of CabHLH18 protein. **(A)** Phylogenetic tree of bHLH proteins in various plant species with bHLH homologs. **(B)** Homology modeling of CabHLH18 protein using MODELLER9.22. **(C)** Molecular dynamics simulation. Backbone of root mean squared deviation (RMSD) plotted versus time (in ps). **(D)** Ramachandran plot analysis. A, B, and L regions: most favored residues; a, b, l, and p regions: additional allowed residues; ~a, ~b, ~l, and ~p regions: generously allowed residues.

A 1,056-bp cDNA sequence from ‘ZHC2’ was amplified by PCR (**Figure 1A**) using LOC107879909-specific primers, and named *CabHLH18*. Bioinformatics analyses revealed that *CabHLH18* is a hydrophilic protein encoding 352 amino acids (**Figure 1B**) and has a bHLH_AtNAI1-like conserved domain at 170–242 at the C-terminus (**Figure 1C**), which mediates endoplasmic reticulum formation and may play a role in plant tolerance to abiotic stress. Phylogenetic tree analyses based on amino acid sequences of various plant species found that CabHLH18 protein had maximum similarity with *Capsicum annuum* XM 016694561.2 protein (**Figure 2A**) and that their domains were similar. The prediction model revealed that the similarity between the 3D structure model of CabHLH18 and the template 5gnj.1.A was as high as 99.6% and that both proteins had a bHLH-binding domain and belonged to the bHLH family, indicating good model quality (**Figure 2B**). The root-mean-square deviation curve reached equilibrium after 2,750 ps, with fluctuations in the range of 1.66–2.24 nm. These results showed that *CabHLH18* had a stable structure (**Figure 2C**). The Ramachandran diagram verification of the protein denoted its suitability because there were no residues in the disallowed regions (**Figure 2D**).

### Subcellular localization of CabHLH18 protein

**Figure 3:**
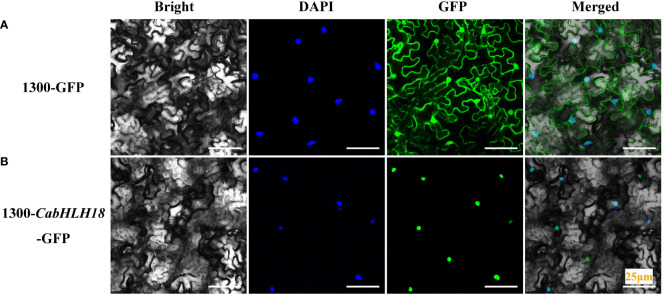
Subcellular localization of CabHLH18 protein in *N. benthamiana* mesophyll cells. **(A)** Vector 1300-GFP was introduced into tobacco leaves. **(B)** Fusion protein 1300-*CabHLH18*-GFP was introduced into tobacco leaves. The sample was observed under a confocal laser-scanning microscope. Green fluorescent protein (GFP), nuclear fluorescence (blue), combined images (green and blue), and bright-field, phase-contrast images are displayed. Bar = 25 µm.

To determine the subcellular localization of CabHLH18 protein, the 1300-*CabHLH18*-GFP fusion protein was expressed transiently in *N. benthamiana* mesophyll cells. The infective solution was injected into *N. benthamiana* from the lower epidermis of the leaves, and the sample was analyzed after 72 h. CabHLH18 protein was localized in the nucleus, while the empty vector GFP signal was distributed throughout the cell, indicating that CabHLH18 might have a regulatory role as a transcription factor (**Figure 3**).

### Transformation and identification of *CabHLH18* gene in hot pepper

**Figure 4:**
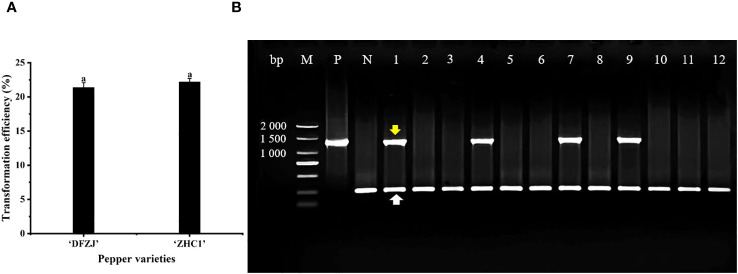
Transformation and identification of *CabHLH18* gene in hot pepper. **(A)** Transformation of *CabHLH18* overexpression vector into the plumules of two pepper cultivars. **(B)** PCR identification of *CabHLH18* transgenic plants. PCR detection of *CabHLH18* gene in the genomic DNA of transgenic T_0_ plant leaves. The yellow arrow indicates the target fragment of the *CabHLH18* gene. The white arrow shows the amplification band of the housekeeping gene *18S*. M: 2,000-bp DNA marker; lanes 1, 4, 7, and 9: transgenic plants; lanes 2, 3, 5, 6, 8, and 10–12: non-transformed plants; P: positive control (*CabHLH18* recombinant plasmid); N: negative control (wild-type DNA).

To investigate the function of *CabHLH18* gene, *CabHLH18* overexpression vector was transformed into the plumules of hot pepper. The specific primers of *CabHLH18* and housekeeping gene *18S* were identified by multiple PCR analyses. The seedlings of hot peppers ‘DFZJ’ and ‘ZHC1’ were selected for transformation, which presented efficiencies of 21.37% and 22.20%, respectively, indicating that this transformation technique was stable and repeatable (**Figure 4A**). The housekeeping primers amplified in all plant samples, indicating that the DNAs were of good quality. Lanes 1, 4, 7, and 9 showed amplification with construct-specific primer sets, indicating that these plants had incorporated the transgene (**Figure 4B**).

### Overexpression of *CabHLH18* gene improved waterlogging tolerance in hot pepper

**Figure 5:**
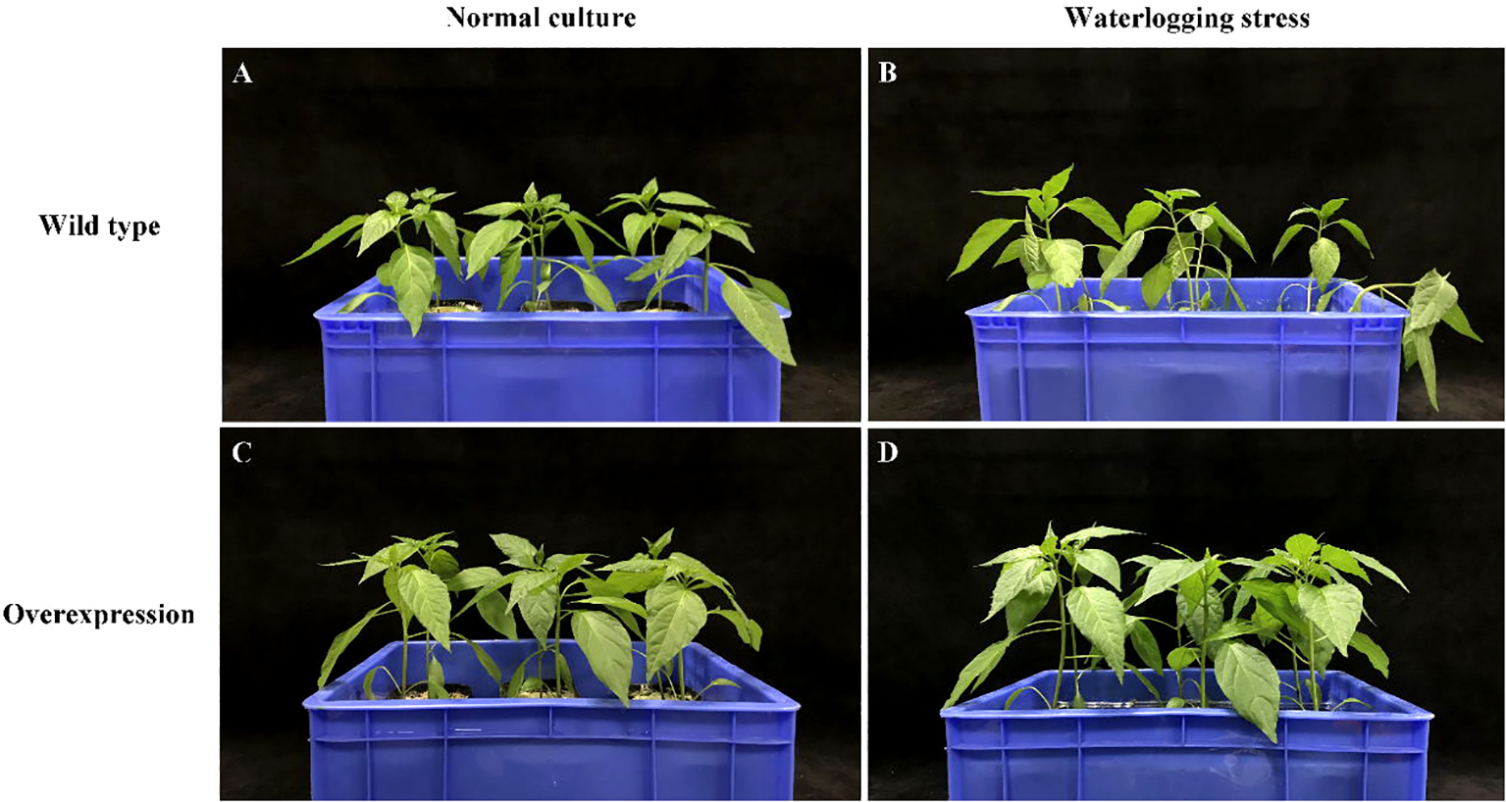
Effects of waterlogging stress on hot pepper ‘DFZJ’ overexpressing *CabHLH18* gene. **(A, B)** Growth of WT pepper under normal and waterlogging stress conditions, respectively. **(C, D)** Growth of overexpression lines under normal and waterlogging stress conditions, respectively.

**Figure 6:**
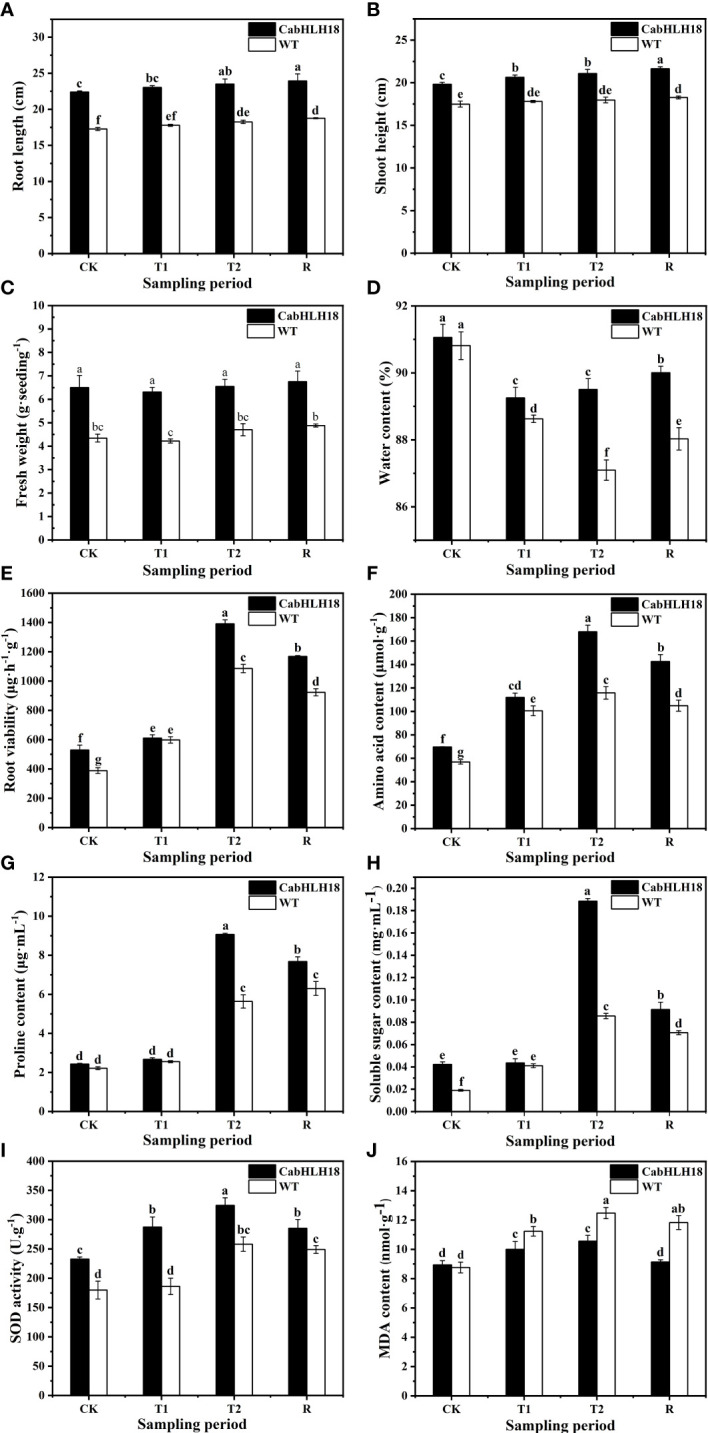
Effects of waterlogging stress and recovery on the **(A)** root length, **(B)** seedling height, **(C)** fresh weight, **(D)** water content, **(E)** root viability, **(F)** amino acid content, **(G)** proline content, **(H)** soluble sugar content, **(I)** SOD activity, and **(J)** MDA content of the overexpression line. CK, T1, T2, and R denote control, 6 h after waterlogging stress, 24 h after waterlogging stress, and 1 h after recovery, respectively. The column represents mean value of SD (n = 3), and values with different letters are significantly different (*p* < 0.05).

After 7 days of waterlogging stress, the leaf wilted degree of hot pepper ‘DFZJ’ overexpression lines was lower than that of the WT plants (**Figure 5**), whereas the root length, seedling height, and fresh weight of the overexpression lines reached 1.29-, 1.17-, and 1.39-fold that of the WT plants at the T2 (24 h after waterlogging stress) stage, respectively (**Figures 6A–C**). The overexpression lines showed greater height and longer roots than the WT plants (**Figures 6A, B**). Under normal culture conditions, no difference was observed in the water content between the overexpression lines and WT plants. However, at the T1 and T2 stages, the water content in the WT plants was less than that in the overexpression lines, showing reductions of 0.70% and 2.69%, respectively. At the R stage, the water content in both the overexpression lines and WT plants increased, and the overexpression lines had higher water content than the WT plants (**Figure 6D**). At the T2 and R stages, the root viability of the overexpression lines was 28.14% and 26.56%, respectively, when compared with the WT plants (**Figure 6E**).

Under waterlogging stress, the amino acid content (**Figure 6F**), proline level (**Figure 6G**), and soluble sugar level (**Figure 6H**) in the roots of the overexpression lines reached a peak at the T2 stage and were 45.03%, 60.67%, and 120.18% higher, respectively, than those in the roots of the WT plants. However, at the R stage, the amino acid content, proline level, and soluble sugar level decreased in the overexpression lines, and the decrease was more rapid than that noted in the WT plants, indicating that the overexpression lines responded more sensitively to waterlogging stress and recovery.

The SOD activity of both the overexpression lines and WT plants increased under waterlogging stress, peaked at the T2 stage, and decreased at the R stage (**Figure 6I**). In particular, the SOD activity of the overexpression lines was higher than that of the WT plants under waterlogging conditions, indicating that the overexpression lines had better ROS scavenging ability. After a longer waterlogging stress period, the MDA content in both the overexpression lines and WT plants increased, reaching a peak at the T2 stage and decreasing at the R stage (**Figure 6J**). However, the MDA content in the overexpression lines was lower than that in the WT plants under both waterlogging stress and recovery conditions, with the overexpression lines presenting greater MDA decline rates than the WT plants after recovery.

## Discussion

### Cloning and expression characteristics of the *CabHLH18* gene

The bHLH family of transcription factors responds to plant abiotic stresses (Kiribuchi et al., 2004; Zhang et al., 2020). A novel bHLH transcription factor, PtrbHLH66, from Triloba orange has been reported to actively regulate plant drought tolerance by root growth effects and ROS clearance (Liang et al., 2022). *Arabidopsis* AtbHLH122 positively regulates plant responses to drought and salt stress by inhibiting *CYP707A3* expression and increasing abscisic acid levels (Liu et al., 2015). Overexpression of *SlbHLH22* in tomato has been observed to increase secondary metabolites and osmoregulatory substances, enhance ROS scavenging ability, and improve drought and salinity tolerance (Waseem et al., 2019). Zhang et al. (2020) identified 122 members of the bHLH transcription factor family in *C. annuum*, among which only a few were noted to regulate plant responses to stresses, such as cold, heat, drought, and salt stress (Zhang et al., 2020). The bHLH transcription factor gene *CabHLH035* in peppers has been found to improve salt tolerance by regulating ion homeostasis and proline biosynthesis (Zhang et al., 2022). Currently, most of the studies related to waterlogging stress on peppers have only concentrated on morphological observation, physiological examination, and biochemical analysis, devoting limited attention to waterlogging tolerance mechanisms (Kato et al., 2020; Kaur et al., 2021; Komatsu et al., 2022). In the present study, the *CabHLH18* gene was cloned from hot pepper ‘ZHC2’ with strong waterlogging tolerance. Phylogenetic tree analyses of bHLH proteins from different plant species showed that CabHLH18 and capsicum-related proteins clustered together, thus suggesting that they might have close genetic relationships in evolution and function.

### Subcellular localization of CabHLH18 protein

A majority of the bHLH transcription factor proteins are located in the nucleus and might have nuclear protein functions. For instance, bHLH122 is localized in the nucleus and plays an important role in drought resistance, osmotic stress resistance, and inhibition of *Arabidopsis* abscisic acid catabolism (Liu et al., 2013). The *ThBHLH1*-encoded protein of the *bHLH* gene of *Salix sphinx* is localized in the nucleus and improves abiotic stress tolerance by increasing osmotic potential and reducing ROS accumulation (Ji et al., 2016). The wheat transcription factor TabHLH39 is located in the nucleus and improves the tolerance of transgenic plants to abiotic stress (Zhai et al., 2016). In the present study, CabHLH18 protein was also localized in the nucleus, indicating that it may predominantly function in the nucleus and might regulate gene expression and control plant responses to waterlogging stress.

### Establishment of the plumules transformation system of hot pepper

The transformation complexity of peppers restricts their development of genetic engineering, breeding, and molecular biology (Mahto et al., 2018). Owing to high genotype dependence and tenacity of pepper (Kothari et al., 2010), it is difficult to achieve a stable transformation system, impeding the development of pepper transgenic technology development (Heidmann and Boutilier, 2015). The transformation of peppers mainly uses *Agrobacterium*-mediated and gene-gun methodologies. For example, cotyledon (Kim et al., 2017) and hypocotyl (Kumar et al., 2012) were used as explants to establish an *Agrobacterium*-mediated transformation system of *Capsicum*; however, this method was unstable and not only required complicated tissue culture processes but also needed specific plant materials to achieve regeneration. Currently, achieving a highly efficient hot pepper transformation system is particularly important, and transgenic plants obtained without tissue culture can significantly reduce the cost. In one study, plant meristems were induced to produce shoots with targeted DNA modifications, and targeted genes were transmitted to the progeny, which sidesteps the need for tissue culture (Maher et al., 2020). Cao et al. (2023) achieved the transformation of several plant species by using a cut–dip–budding delivery system to transform plant genes without tissue culture (Cao et al., 2023). In our study, the seed coats of hot pepper were removed to expose the plumules and then infected with *Agrobacterium* containing the target gene. Effective transformation of peppers could be achieved with radicle length of 1–2 mm, *Agrobacterium* density (OD_600_) of 0.50–0.60 nm, at 15 kPa. Two pepper cultivars (‘ZHC1’ and ‘DFZJ’) were transformed by this method, and no significant difference was found in the transformation efficiency, indicating that the transformation system was stable in the hot peppers. This transformation method does not depend on tissue culture, thus avoiding some problems associated with tissue culture such as contamination, browning, and soma clonal variation, and the efficient transformation system might provide strong technical support for pepper genetics and breeding studies.

### Effect of the *CabHLH18* gene on waterlogging tolerance of transgenic peppers

Waterlogging stress inhibits root respiration and ATP synthesis, blocking the generation of water potential gradients and ion transport systems on the root endodermis and causing plant withering (Sairam et al., 2008). Under well-watered conditions, the overexpression of the *Populus euphratica* gene, *oxPebHLH35*, in *Arabidopsis* resulted in longer taproots, higher leaf numbers, and increased leaf area, thus improving the plant water stress tolerance, when compared with the vector control plants (Waseem et al., 2019). After treatment with 100 mM and 150 mM NaCl, the roots of the *CabHLH035* transgenic tobacco lines were longer than those of the WT plants. In comparison with the WT plants, the *CabHLH035* transgenic lines had substantially lower water loss (Zhang et al., 2022). In our study, under waterlogging stress, the WT pepper plants exhibited higher leaf wilting degree and lodging at the T2 stage and had lower water content, when compared with those noted in the overexpression lines. Furthermore, the seedlings of the overexpression lines were stronger than the WT plants under the same growth conditions (**Figures 6A–E**). These results are similar to those reported in a previous study (Waseem et al., 2019; Zhang et al., 2022). Thus, under waterlogging stress, the growth of the WT pepper plants was inhibited, whereas the overexpression lines adapted to this stress and maintained normal growth.

Plants can adapt to waterlogging stress through the accumulation of proline and soluble sugar (Nanjo et al., 1999; Hildebrandt et al., 2015; Hildebrandt, 2018; Khan et al., 2020). Proline is a main solute molecule involved in plant osmotic regulation and is also a free radical scavenger, protecting the plant’s photosynthetic activity and cells from damage, to ensure sustained plant growth under long-term stress (Silva-Ortega et al., 2008; Kavi Kishor and Sreenivasulu, 2014). The increase in higher proline content regulates the osmotic potential and improves abiotic stress tolerance (Ji et al., 2016). The *ThbHLH1* gene has been reported to activate proline biosynthesis by inducing the expression of *P5CS* and *BADH/ALDH* (Ji et al., 2016). Overexpression of *VvbHLH1* in *Arabidopsis* has been found to increase the proline content, maintain osmotic balance between intracellular and extracellular environments, and protect membrane integrity, thus enhancing salt and drought tolerance (Wang et al., 2016). Furthermore, *TabHLH39* transgenic plants have been noted to exhibit higher levels of soluble sugars and proline and lower levels of electrolyte leakage. The TabHLH39 protein in the transgenic plants can protect the plant cells by increasing the soluble sugar content to provide energy and redistribute the soluble osmotic sugars and improve plant stress resistance by promoting proline accumulation. The soluble sugars and proline act as osmotic regulators and molecular chaperones to protect the protein integrity and enhance enzyme activity, thus improving *Arabidopsis* resistance to abiotic stress (Zhai et al., 2016). The synthesis of soluble sugars and other substances can provide sufficient reducing sugars under waterlogging stress (Sairam et al., 2009). In the present study, the contents of proline, soluble sugar, and amino acid levels in *CabHLH18* overexpression lines were significantly higher than those in the WT plants under waterlogging stress. The overexpression lines accumulated more substances involved in osmotic regulation and energy supply, leading to stronger osmotic regulation ability and adequate energy supply, which mitigated the damage caused by waterlogging stress.

Limited oxygen levels can cause ROS accumulation under waterlogging stress, resulting in membrane lipid peroxidation, structural changes in proteins and nucleic acids (Mittler et al., 2004; Bansal and Srivastava, 2012; Mittler, 2017), decreased activity of antioxidant enzymes, and increased MDA. Plants maintain ROS homeostasis to adapt to abiotic stress by activating the antioxidant system, and the increased SOD activity can enhance plant resistance (Tavanti et al., 2021). Zhang et al. (2022) reported CabHLH035-protected plants from oxidative damage by removing ROS through increased expression of the SOD gene (Zhang et al., 2022). In the present study, SOD activity was higher, and MDA content was lower in the overexpression lines, when compared with those in the WT plants under waterlogging stress, indicating that *CabHLH18* overexpression lines could reduce the damage caused by ROS under waterlogging stress.

## Conclusion

In this study, the *CabHLH18* gene was found to contain a 1,056-bp ORF and encode 352 amino acids. The CabHLH18 protein was determined to be located in the nucleus. Subsequently, an effective transformation system of hot pepper was established, with an efficiency of 22.20%. Under waterlogging stress, *CabHLH18* overexpression lines showed significantly greater root length, plant height, fresh weight, water content, and root viability, when compared with WT plants. The contents of amino acids, proline, soluble sugars, and SOD activity were also significantly higher, but the MDA level was lower in the overexpression lines, when compared to the WT plants. Thus, the *CabHLH18* gene could enhance waterlogging tolerance of *CabHLH18*-overexpressing hot pepper and might be a valuable gene for improving waterlogging tolerance of crops.

## Data availability statement

The datasets presented in this study can be found in online repositories. The names of the repository/repositories and accession number(s) can be found in the article/**Supplementary Material**.

## Author contributions

HT: Software, Writing – original draft, Data curation, Investigation. GF: Writing – original draft, Investigation. XX: Investigation, Writing – original draft. HW: Writing – original draft, Data curation. SZ: Conceptualization, Writing – review & editing. GG: Writing – review & editing, Conceptualization, Funding acquisition.
